# Resident *Prrx1* lineage stromal cells promote T cell survival in the spleen

**DOI:** 10.1093/jmcb/mjy073

**Published:** 2019-01-08

**Authors:** Wanyao Zhang, Qian Yu, Huijuan Liu, Baojie Li

**Affiliations:** Bio-X Institutes, Key Laboratory for the Genetics of Developmental and Neuropsychiatric Disorders, Ministry of Education, Shanghai Jiao Tong University, Shanghai, China

**Dear Editor,**

Spleen is a secondary lymphoid organ (SLO). It is composed of white pulps, lymph node-like structures that contain T and B cells, and red pulps, which filter effete red blood cells. The architecture of spleen is supported by stromal cells that can be classified into at least four subtypes based on expression of CD31 and podoplanin (gp38): lymphatic endothelial cells (LECs, CD31^+^gp38^+^), blood endothelial cells (BECs, CD31^+^gp38^−^), fibroblastic reticular cells (FRCs, CD31^−^gp38^+^), and double-negative stromal cells (DNSCs, CD31^−^gp38^−^) (Link et al., 2007). The stromal cells form a physical framework that supports compartmentation of SLOs (Astarita et al., 2015). In addition, growing evidence indicates that stromal cells also play critical roles in immune cell homeostasis (Mueller and Germain, 2009). Stromal cells in SLOs can secrete cytokines, e.g. IL7, CCL19, and CCL21, to regulate proliferation, apoptosis, and migration of immune cells (Roozendaal and Mebius, 2011).

Yet the identities and functions of these stromal subtypes remain not well understood. Previous studies have shown that *Nkx2-5*^+^*Islet1*^+^ mesenchymal cells produce FRCs, FDCs, MRCs, and mural cells, but not DNSCs (Castagnaro et al., 2013). In this study, we identified *Prrx1* as a marker for DNSCs in the spleen and provided evidence that these stromal cells promote T cell survival via secreting IL6.

We have previously used a transgenic *Prrx1-Cre* mouse line to label bone marrow mesenchymal stem (stromal) cells (Cong et al., 2017; Wu et al., 2017). Our lineage tracing experiments revealed that the *Prrx1* lineage cells were also present in the spleen. *Prrx1* lineage cells were predominantly localized in the white pulp and the marginal zone of spleen (Figure 1A). Immuno-staining of spleen sections revealed that *Prrx1* lineage stromal cells were negative for CD31 and gp38 (Figure 1A), suggesting that they represent DNSCs. Flow cytometry analysis of CD45^−^Ter119^−^ spleen stromal cells confirmed this finding (Supplementary Figure S1). These results suggest that *Prrx1* mainly marks DNSCs in the spleen.

**Figure 1:**
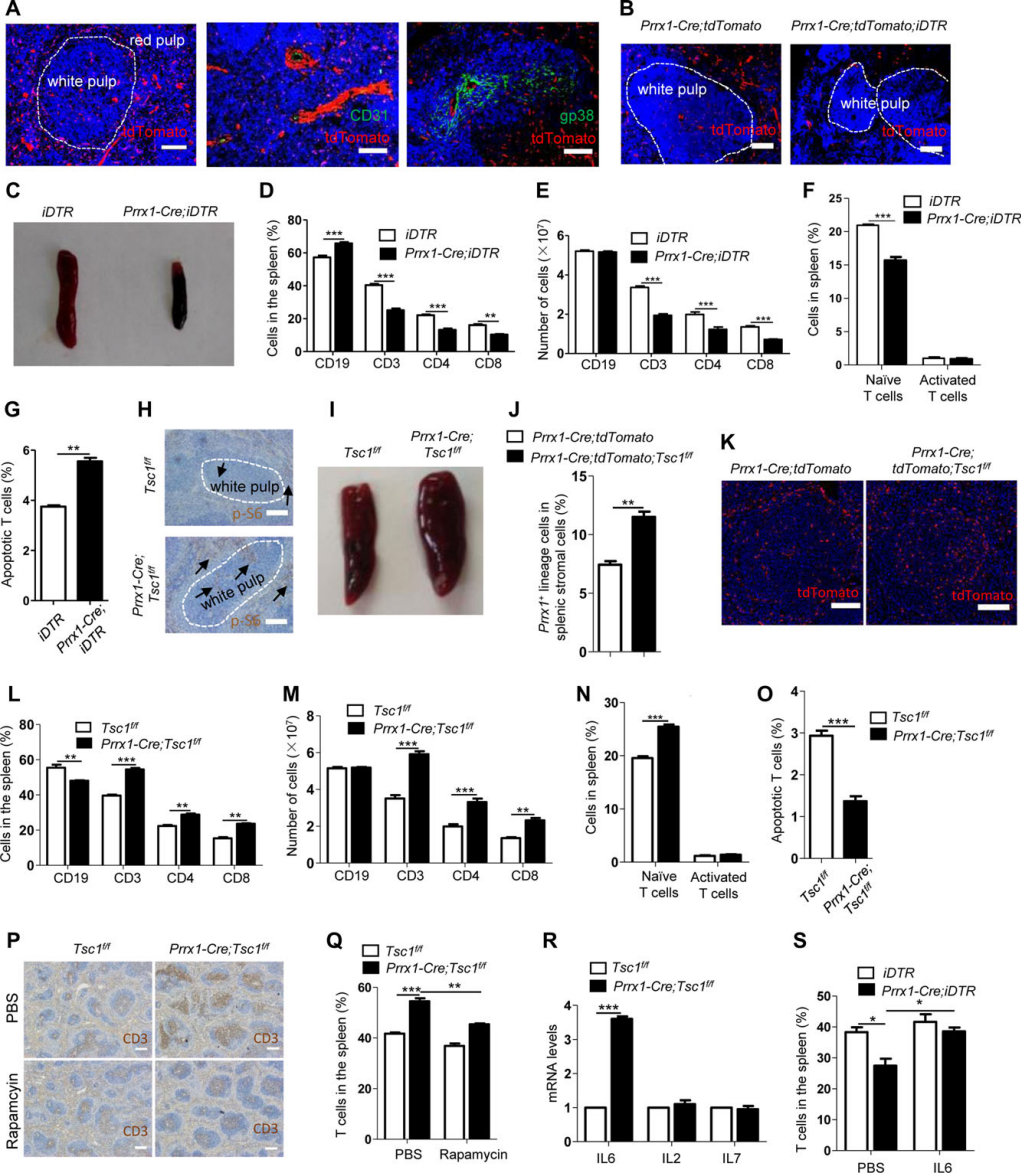
Resident *Prrx1* lineage stromal cells promote T cell survival via IL6 in the spleen. (**A**) Location and characterization of *Prrx1* lineage cells of the spleen of 8-week-old *Prrx1*-*Cre*;*tdTomato* mice. Spleen sections were stained with anti-CD31 and anti-gp38 antibodies, respectively. (**B**–**G**) Specifically killing *Prrx1* lineage cells resulted in decreased number of *Prrx1* lineage cells in the spleen (**B**), decreased spleen size (**C**), decreased percentage and number of T cells (**D** and **E**), decreased percentage of naïve T cells (**F**), and increased number of apoptotic T cells (**G**). CD3^+^ T cells were stained with annexin V and 7-AAD. (**H–O**) Ablation of *Tsc1* in *Prrx1* lineage cells resulted in stronger p-S6 signals shown by arrows (**H**), increased spleen size (**I**), increased number of *Prrx1* lineage cells (**J** and **K**), increased number and percentage of T cells (**L** and **M**), increased percentage of naïve T cells (**N**), and decreased number of apoptotic T cells (**O**). (**P** and **Q**) Rapamycin could rescue the increase in T cells in *Prrx-Cre*;*Tsc1*^*f/f*^ mice. Sections were stained with CD3 antibody (**P**) or spleen cells were analyzed using flow cytometry (**Q**). (**R**) IL6 mRNA level was increased after *Tsc1* ablation in *Prrx1* lineage cells. *Prrx1* stromal cells were collected by FACS sorting and mRNA level was analyzed by quantitative PCR. (**S**) Injection of IL6 increased the number of T cells in *Prrx1*;*iDTR* mice. Spleen T cells were analyzed using flow cytometry. Scale bar, 100 μm (**A**, **B**, **H**, **K**) or 250 μm (**P**). *n* = 8 (**D**–**F**) or 6 (**G**, **J**, **L**–**O**, **R**, **S**).

We isolated *Prrx1* lineage cells by FACS sorting from spleens of *Prrx1-Cre*;*tdTomato* mice for further analysis. We analyzed the expression of MSC markers and found that *Prrx1* lineage cells expressed Sca-1, but not CD105 or CD73 (Supplementary Figure S2A). Besides, these cells adhered to culture dishes, proliferated, and could differentiate into osteoblasts, chondrocytes, or adipocytes *in vitro* (Supplementary Figure S2B). Previous studies have shown that the mixed spleen stromal cultures also have these MSC features.

To determine the *in vivo* functions of *Prrx1* lineage stromal cells in spleen, we crossed *Prrx1*-*Cre* mice to *ROSA-STOP-iDTR* mice, which have a floxed STOP codon in front of diphtheria toxin receptor (DTR) open reading frame (Buch et al., 2005). Expression of Cre in *Prrx1* lineage cells would delete the STOP codon, leading to expression of iDTR. Injection of diphtheria toxin (DT) killed the *Prrx1* lineage cells (Figure 1B). These mice showed a significant reduction in the size of spleen, although the architecture was not obviously disrupted (Figure 1C and Supplementary Figure S3), suggesting that the *Prrx1* lineage stromal cells do not play a structural role in the spleen.

We also found that the percentage and the number of T cells including naïve T cells, but not B cells or activated T cells, were significantly decreased in the spleen when *Prrx1* lineage cells were depleted (Figure 1D–F), accompanied by an increase in apoptotic T cells but not proliferating T cells (Figure 1G and Supplementary Figure S4). These results suggest that one function of the *Prrx1* lineage stromal cells is to promote T cell survival in the spleen.

We then took advantage of the *Prrx1*-*Cre*-mediated knockout mouse lines in our laboratory, including *Jag1*, *Tsc1*, and *p38α* (Cong et al., 2017; Wu et al., 2017), to test whether ablation each of these genes in *Prrx1* lineage stromal cells affected the spleen. We found that *Prrx1-Cre*;*Jag1*^*f/f*^ mice and *Prrx1-Cre*;*p38α*^*f/f*^ mice did not display any significant change in the architecture or numbers of T and B cells in spleen (Supplementary Figures S5 and S6). However, ablation of *Tsc1* in *Prrx1* lineage cells of *Prrx1-Cre*;*Tsc1*^*f/f*^ mice resulted in stronger p-S6 signals (Figure 1H and Supplementary Figure S7) indicating mTOR activation, an enlarged spleen (Figure 1I), and an increase in the number of *Prrx1* lineage cells in the spleen (Figure 1J and K). Furthermore, the number and percentage of T cells including naïve T cells were increased (Figure 1L–N). Yet, the numbers of T cells in inguinal lymph nodes (iLNs), thymus, bone marrow, and blood were not significantly affected (Supplementary Figure S8). We further showed that the spleen phenotypes were associated with a decrease in apoptotic T cells but without change in proliferating T cells in the spleen (Figure 1O and Supplementary Figure S9). Moreover, rapamycin, an inhibitor of mTORC1 signaling, could rescue the spleen phenotypes (Figure 1P and Q), suggesting that these phenotypes were caused by enhanced mTORC1 activation.

It is known that stromal cells secrete cytokines to regulate immune cells in SLOs. We then analyzed the levels of these cytokines in spleen stromal cells isolated from *Prrx1-Cre*;*Tsc1*^*f/f*^ and control mice. The mRNA levels of IL2 and IL7, which play vital roles in the survival of T cells, and CCL19 and CCL21, which recruit T cells into SLOs, were all not affected by *Tsc1* ablation (Figure 1R and Supplementary Figure S10). However, the IL6 level was significantly increased in *Tsc1*^−/−^ spleen stromal cells (Figure 1R). It has been previously shown that IL6 could promote T cell survival *in vitro* (Xu et al., 2007). We injected IL6 into *Prrx1-Cre*;*iDTR* mice that received DT and found that IL6 rescued the decrease in the number of T cells in the spleen, which was attributable to increased T cell apoptosis (Figure 1G). Yet, IL6 showed no effect on spleen T cells in wild-type mice (Figure 1S). These results suggest that *Prrx1*^+^ stromal cells-secreted IL6 helps to prevent T cells from death in the spleen.

We also examined *Prrx1* lineage cells in inguinal lymph node and found that *Prrx1* lineage cells mainly labeled DNSCs and some BECs (Supplementary Figure S11A–C). These cells, like spleen stromal cells, also showed MSC features (Supplementary Figure S2 and data not shown). Depletion of *Prrx1*^+^ lineage cells also led to decreases in the number of *Tomato*^+^ cells, lymph node size, and the percentage of T cells in iLNs (Supplementary Figure S11D–G), accompanied by an increase in apoptotic T cells but not proliferating T cells (Supplementary Figure S11H and I). However, *Tsc1* ablation in *Prrx1* lineage did not affect the number of T cells in iLNs (Supplementary Figure S8), nor did IL6 injection rescue the decrease of T cells in iLNs of mice depleted of *Prrx1* lineage cells (Supplementary Figure S11J). These results suggest that other mechanisms may exist in lymph nodes in regulating T cell survival.

In summary, we have identified a genetic marker for DNSCs in the spleen. Our cell depletion and gene ablation experiments show that the *Prrx1*-marked stromal cells secrete IL6 to promote T cell survival in the spleen. Interestingly, in the bone marrow, *Prrx1* lineage stromal cells have been shown to act as a niche for hematopoietic stem cells (Greenbaum et al., 2013). Thus, *Prrx1* lineage cells may play important but distinct roles in primary and secondary immune organs. [*Supplementary material is available at Journal of Molecular Cell Biology online*.]

## Supplementary Material

Supplementary Data
