# Supplementary material for: Resident Prrx1 lineage stromal cells promote T cell survival in the spleen
Source: J Mol Cell Biol. 2019 Jan 8;11(2):182–4. doi: 10.1093/jmcb/mjy073 (PMC6392099; doi:10.1093/jmcb/mjy073)
Supplement: Supplementary Data [file mjy073_supplementary_material.pdf]

Supplementary Materials and methods

Mice

All mice were maintained under pathogen-free conditions in the animal center of Shanghai Jiao Tong University. The mouse protocols were approved by the animal research committee of Shanghai Jiao Tong University. Transgenic *Prrx1-Cre* mice, *ROSA-tdTomato* mice, *Tsc1*$^{f/f}$ mice, and *Jag1*$^{f/f}$ mice were purchased from Jackson Laboratory. *p38α*$^{f/f}$ mice were from Yibin Wang Lab. *iDTR* mice were from Waisman Lab.

Isolation of stromal cells

Spleens and inguinal lymph nodes were dissected and cut into small pieces and then incubated in 1.5 ml of RPMI medium 1640 containing 0.1 mg/ml DNase Ⅰ (Roche), 0.8 mg/ml Dispase (Roche), 1.25 mg/ml collagenase D (Roche), and 0.2 mg/ml collagenase P (Roche) at 37 ℃ for 45 min with constant shaking. The cell suspension was gently passed through a 24-gauge needle twice and washed with PBS containing 0.5% BSA and 2 mM EDTA. Hematopoietic cells were depleted with anti-CD45 and anti-Ter119 microbeads (Miltenyi Biotec).

Flow cytometry

Cells were stained with fluorescent dye-conjugated antibodies. Hematopoietic cells were gated out with anti-Ter119 and CD45 antibodies. Intracellular staining was performed using Cell Fixation and Permeabilization reagents (BD). Samples were incubated with the antibodies: CD45-PE-Cy7, Ter119-PE-Cy7, gp38-APC, CD31-FITC for the stromal cells subsets, Fibroblast Reticular cells (FRC, $CD45^-Ter119^-CD31^-gp38^+$), Blood Endothelial cells (BEC, $CD45^-Ter119^-CD31^+gp38^-$), Lymphoid Endothelial cells (LEC, $CD45^-Ter119^-CD31^+gp38^+$), double negative stromal cells (DNSCs, $CD45^-Ter119^-CD31^-gp38^-$); CD3-PE, CD4-APC-Cy7, CD8-PE-Cy7, CD19-FITC for T ($CD3^+$, $CD3^+CD4^+$, $CD3^+CD8^+$) and B ($CD19^+$) cells respectively; CD3-FITC, CD4-Pacific blue, CD25-APC, CD44-APC-Cy7, CD62L-PE-Cy7 for naïve T cells

($CD3^+CD4^+CD25^-CD62L^{hi}CD44^{low}$); CD3-FITC, CD4-Pacific blue, CD69-PE for activated T cells ($CD3^+CD4^+CD69^+$); CD3-APC-Cy7, Annexin V-FITC, and 7-AAD (or PI) for T cells apoptosis; CD3-APC-Cy7, Ki67-FITC for T cells proliferation; Sca-1-FITC, CD106-FITC, CD105-FITC, CD73-FITC, CD44-FITC, CD29-FITC, CD11b-FITC for MSCs. All these antibodies were from Biolegend. Stained cells were analyzed with a BD LSRFortessa™ X-20 (BD Biosciences) and FlowJo software. Cell sorting was performed using a FACSAriaIII (BD Biosciences).

Cell culture

Stromal cells are cultured in 6 well plates in α-MEM containing 10% FBS, 100 U/ml penicillin, and 100μg/ml streptomycin, at 37 ℃ in 5% CO_2 atmosphere. After 72 hours, non-adherent cells were removed and the medium was changed. When adherent cells were 70-80% confluent, they were split and expanded into larger flasks.

MSC differentiation assay

Adipocyte differentiation was achieved after a 3-week culture of MSCs with adipogenic medium containing 10^{-6} M dexamethasone, 10 μg/ml insulin, and 100 μg/ml 3-isobutyl-1-methyl-xantine (all from Sigma). Osteoblast differentiation was achieved after 2-weeks culture with osteogenic medium containing 50 μg/ml ascorbic acid, 10^{-7}M dexamethasone, and 10 mM β-glycerophosphate. Chondrocyte differentiation was achieved after 3-week culture with chondrogenic medium containing 10^{-7} M dexamethasone, 10 ng/ml TGF-β, 50 μg/ml ascorbic acid, 40 μg/ml proline and ITS^+ Premix. Oil Red O, alkaline phosphatase, and Alcian Blue were used to stain for adipocytes, osteoblasts, and chondrocytes respectively.

Histology and Immunohistochemistry

Spleens were fixed in 4% PFA in PBS and embedded in paraffin. For frozen sections, spleens were embedded in OCT compound. Spleen sections were prepared by Leica CM 3050S. After blocking, samples were incubated with the antibodies:

CD31 (Abcam), gp38 (Biolegend), CD3 (Abcam), and p-S6 (CST) respectively. All images were taken with microscope (Nikon ECUPSE 80*i*).

Quantitative PCR

Total RNA was extracted from sorted cells using Trizol (Invitrogen). cDNA was synthesized with random primers and amplified in duplicate by QuantiTect SYBR Green PCR kit (Roche) using an ABI 7500 sequence detector (Applied Biosystems). PCR efficiency was normalized using cDNA from WT mice. Primer sequences were as following:

IL2-forward:TGAACTTGGACCTCTGCG

IL2-reversed:AGGGCTTGTTGAGATGATGC

IL6-forward:GTTGCCTTCTTGGGACTGATG

IL6-reversed:GACTCTGGCTTTGTCTTTCTTGTT

IL7-forward:GATAGTAATTGCCCGAATAATGAACCA

IL7-reversed:GTTTGTGTGCCTTGTGATACTGTTAG

CCL19-forward:CTGCCTCAGATTATCTGCCAT

CCL19-reversed:AGGTAGCGGAAGGCTTTCAC

CCL21-forward:AAGGCAGTGATGGAGGGG

CCL21-reversed:CGGGGTAAGAACAGGATTG

Administration of rapamycin, DT and IL6

For rapamycin administration, rapamycin was i.p. injected daily for 14 days at 3 mg/day/g body weight. For DT administration, DT was i.p. injected daily for 5 days at 5 ng/day/g body weight. For IL-6 administration, we firstly treated mice with DT for 2 days; and then IL-6 was i.v. injected at 2.5 ng/day/g body weight with DT for 3 more days.

Statistics analysis

Data are expressed as mean ± SEM. An unpaired Student's t-test was used to

analyze data sets between two groups. *$P<0.05$, **$P<0.01$, and ***$P<0.001$ indicated a significant difference. Statistical calculations were performed using GraphPad Prism.

Supplementary Figure legends

Supplementary Figure S1. Characterization of *Prrx1* lineage cells with flow cytometry. tdTomato-labeled *Prrx1* lineage cells of the spleen were analyzed for the expression of CD31 and gp38 with flow cytometry.

Supplementary Figure S2. *Prrx1* lineage cells of the spleen showed characteristics of MSCs. (A) *Prrx1* lineage cells from spleen were analyzed with flow cytometry for expression of MSC markers. **(B)** Tri-lineage differentiation of *Prrx1* lineage cells isolated from the spleen. Osteoblasts were stained for ALP, adipocytes were stained with Oil Red, and chondrocyte were stained with Alcian Blue.

Supplementary Figure S3. The architecture of the spleen was not altered when *Prrx1* lineage stromal cells were depleted. H/E staining revealed normal architecture of the spleen following DT injection for 5 days. Scale bar, 100 μm.

Supplementary Figure S4. Proliferation of T cells was not altered in *Prrx1* lineage stromal cells depleted mice. Tomato-labeled *Prrx1* lineage stromal cells were stained for Ki67 and then analyzed with flow cytometry. The results are representative of three independent experiments.

Supplementary Figure S5. Ablation of *Jag1* in *Prrx1* lineage cells did not significantly affect the architecture or the numbers of T or B cells in the spleen. (A) H/E staining of spleen sections revealed no gross structural change in *Prrx1-Cre*; $Jag1^{f/f}$ mice. **(B)** Flow cytometry analysis revealed no alteration in the numbers of T or B cells. N=6.

Supplementary Figure S6. Ablation of *p38α* in *Prrx1* lineage cells did not significantly affect the architecture or the numbers of T or B cells in the spleen. (A) H/E staining of spleen sections revealed no gross structural change in *Prrx1-Cre*; $p38\alpha^{f/f}$ mice. **(B)** Flow cytometry analysis revealed no alteration in the numbers of T or B cells. N=6.

Supplementary Figure S7. *Tsc1* ablation led to an incease in p-S6 signals. FACS sorted *Prrx1* lineage cells from the spleen were analyzed by Western blotting.

Supplementary Figure S8. *Prrx1-Cre*; *Tsc1*$^{f/f}$ mice showed normal numbers of T cells in other tissues. The numbers of T cells from inguinal lymph nodes, thymus, bone marrow and blood were analyzed with flow cytometry. N=6.

Supplementary Figure S9. Proliferation of T cells in the spleen of *Prrx1-Cre*; *Tsc1*$^{f/f}$ mice showed no alteration. N=6.

Supplementary Figure S10. mRNA levels of CCL19 and CCL21 were not affected after ablation of *Tsc1* in *Prrx1* lineage cells. *Prrx1* lineage cells were collected by flow cytometric sorting, and then levels of mRNA were analyzed by quantitative PCR. N=5.

Supplementary Figure S11. The function of Prrx1 lineage stromal cells in inguinal lymph node. (A) Location of *Prrx1* lineage cells in the inguinal lymph node of 8-weeks-old *Prrx1-Cre*; *tdTomato* mice. **(B)** Characterization of *Prrx1* lineage cells. Inguinal lymph node sections were stained with anti-CD31 and anti-gp38 antibodies respectively. Scale bar, 100 μm. **(C)** Characterization of *Prrx1* lineage cells. tdTomato labeled *Prrx1* lineage cells of inguinal lymph nodes were analyzed for expression of CD31 and gp38 with flow cytometry. **(D)** Specific killing *Prrx1* lineage cells resulted in a decrease in the number of *Prrx1* lineage cells in lymph node. **(E)** Specific killing *Prrx1* lineage cells resulted in a decrease in lymph node size. **(F)** Specific killing *Prrx1* lineage cells resulted in a decrease in the percentage of T cells, N=6. **(G)** Specific killing *Prrx1* lineage cells resulted in a decrease in the percentage of naïve T cells, N=6. **(H)** Proliferation of T cells was not altered in *Prrx1* lineage stromal cells-depleted mice. Tomato-labeled *Prrx1* lineage stromal cells were stained for Ki67 and then analyzed with flow cytometry, N=3. **(I)** Specific killing *Prrx1* lineage cells resulted in an increase in the number of apoptotic T cells. CD3^{+} T cells were stained with Annexin V and 7-AAD, N=6. **(J)** Injection of IL6 did not rescue the T cells number of *Prrx1*; *iDTR* mice. Inguinal lymph node T cells were analyzed using flow cytometry, N=6.

Figure S1

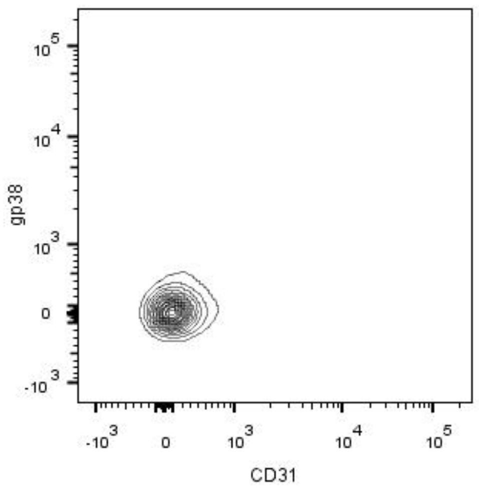

Figure S2

A

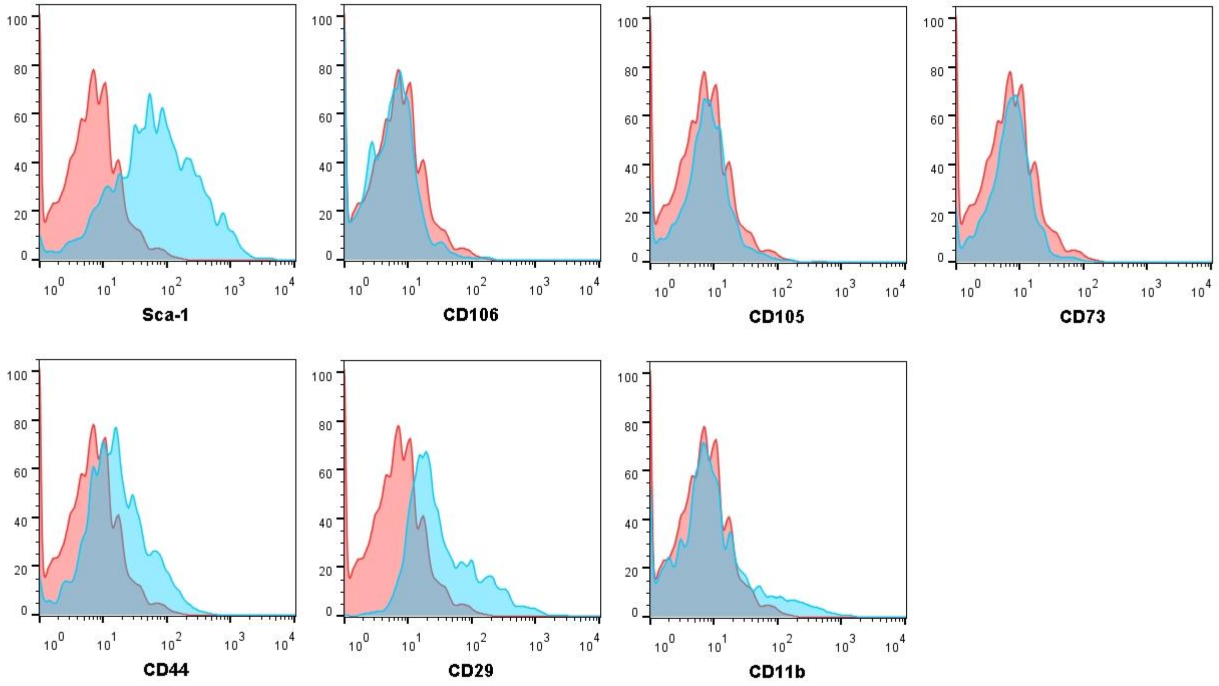

B

ALP Oil Red Alcian Blue

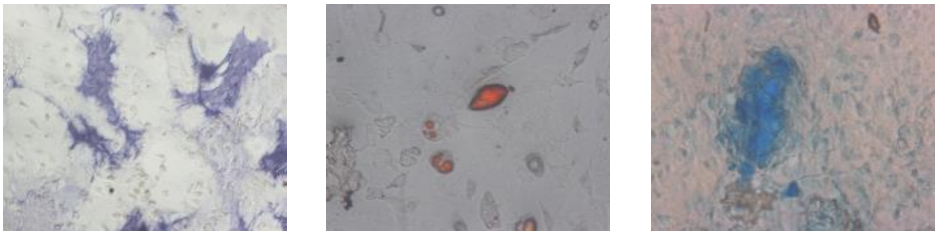

Figure S3

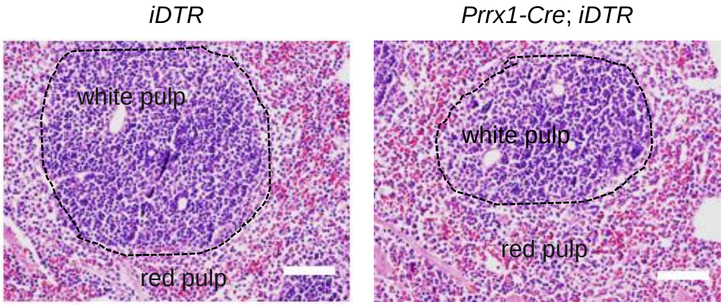

Figure S4

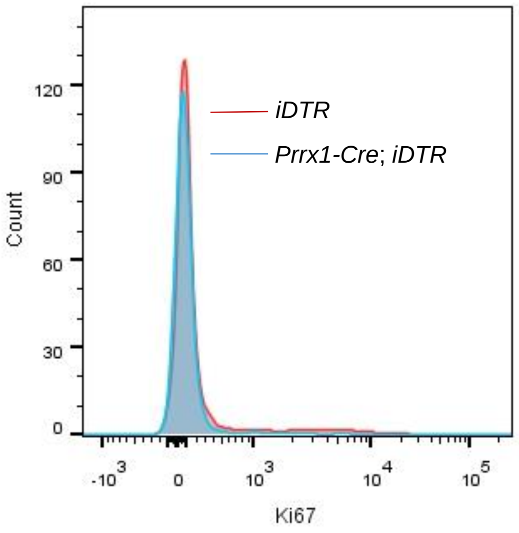

Figure S5

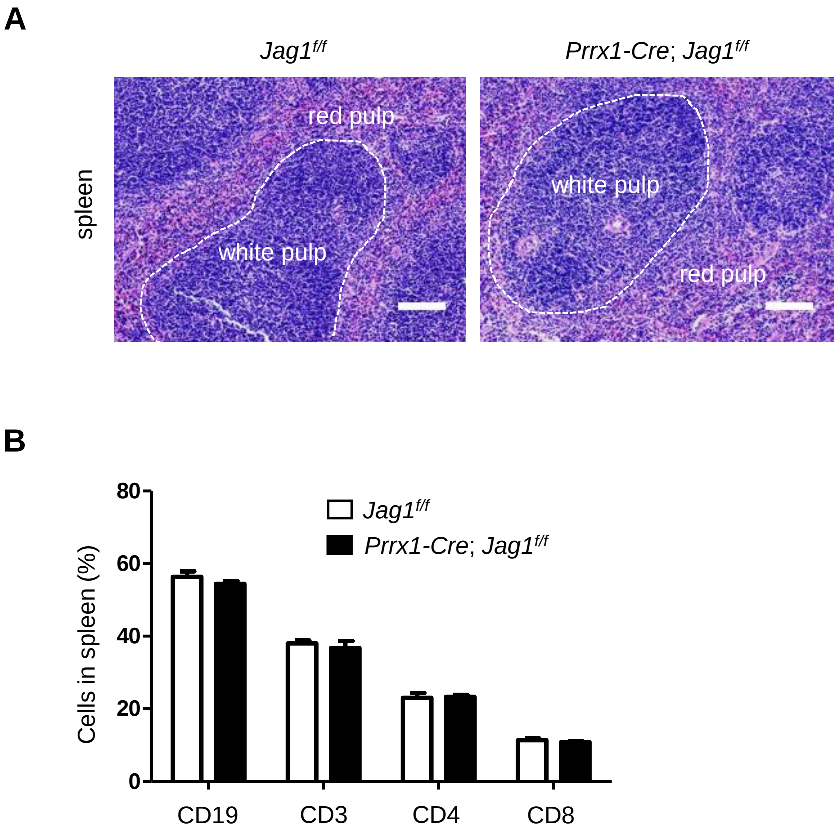
A
Jag1f/f
Prrx1-Cre; Jag1f/f
spleen
red pulp
white pulp
white pulp
red pulp
B
80
60
40
20
0
Cells in spleen (%)
Jag1f/f
Prrx1-Cre; Jag1f/f
CD19
CD3
CD4
CD8

Figure S6

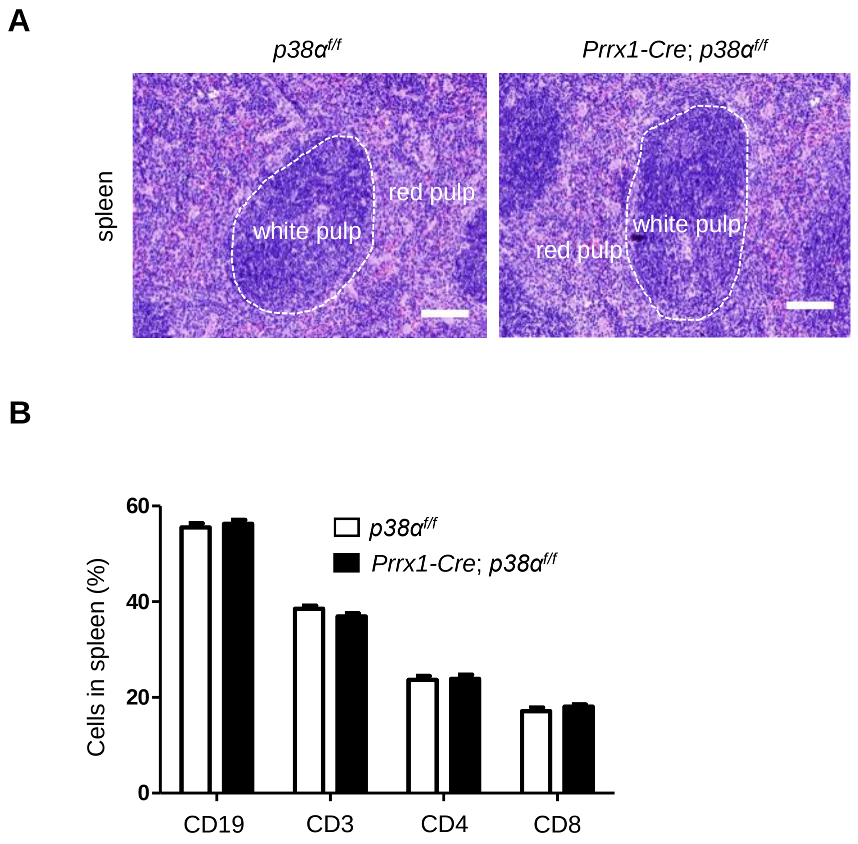
A
p38α f/f
Prrx1-Cre; p38α f/f
spleen
white pulp
red pulp
red pulp
white pulp
B
60
40
20
0
Cells in spleen (%)
p38α f/f
Prrx1-Cre; p38α f/f
CD19
CD3
CD4
CD8

Figure S7

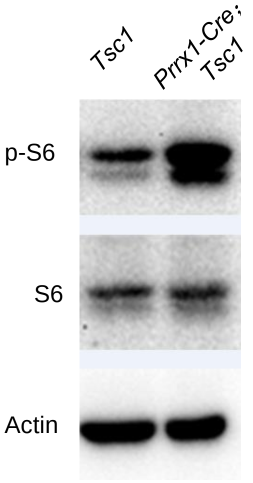

Figure S8

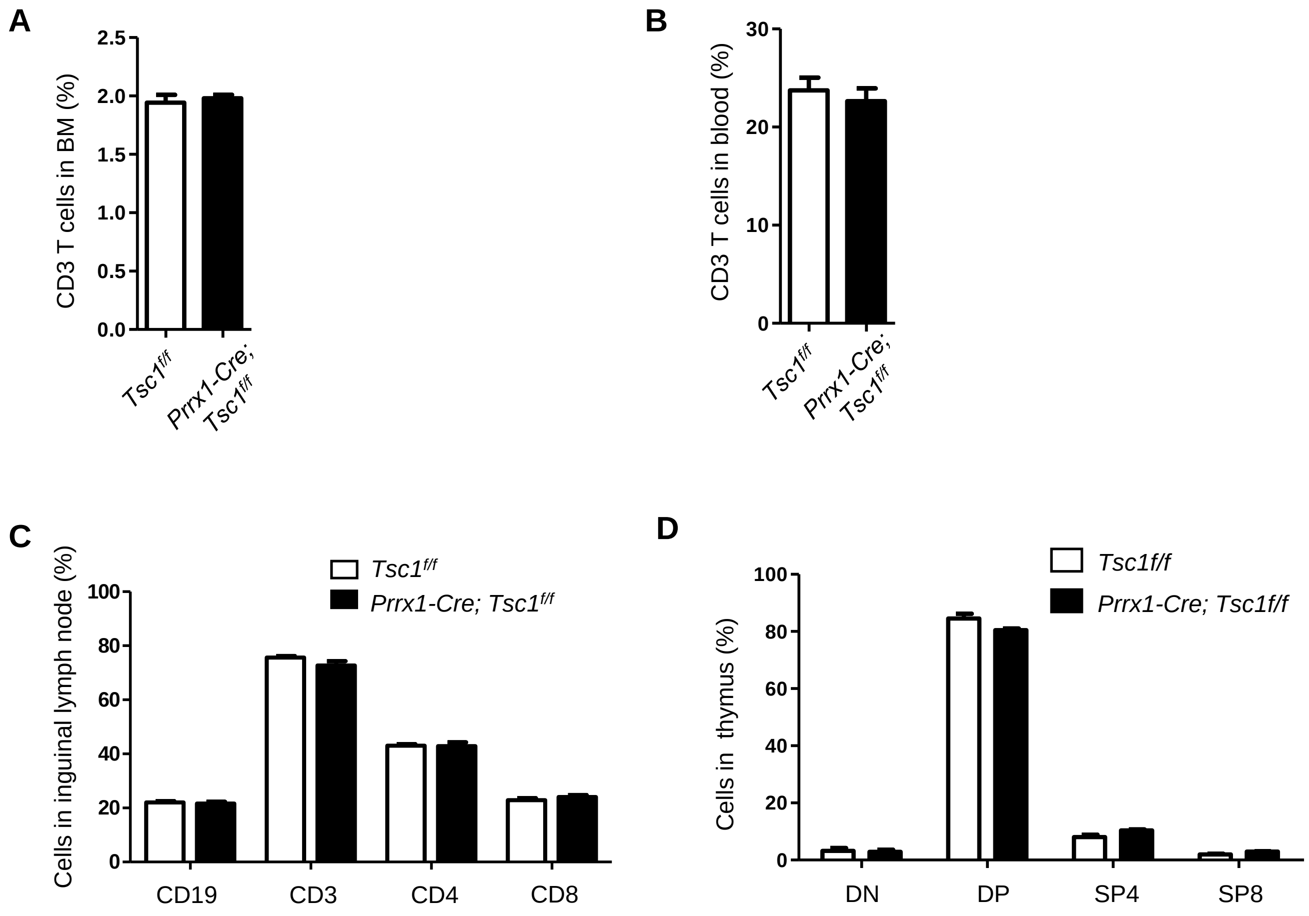

A
CD3 T cells in BM (%)
2.5
2.0
1.5
1.0
0.5
0.0
Tsc1f/f
Prrx1-Cre; Tsc1f/f
B
CD3 T cells in blood (%)
30
20
10
0
Tsc1f/f
Prrx1-Cre; Tsc1f/f
C
Cells in inguinal lymph node (%)
Tsc1f/f
Prrx1-Cre; Tsc1f/f
100
80
60
40
20
0
CD19
CD3
CD4
CD8
D
Cells in thymus (%)
Tsc1f/f
Prrx1-Cre; Tsc1f/f
100
80
60
40
20
0
DN
DP
SP4
SP8

Figure S9

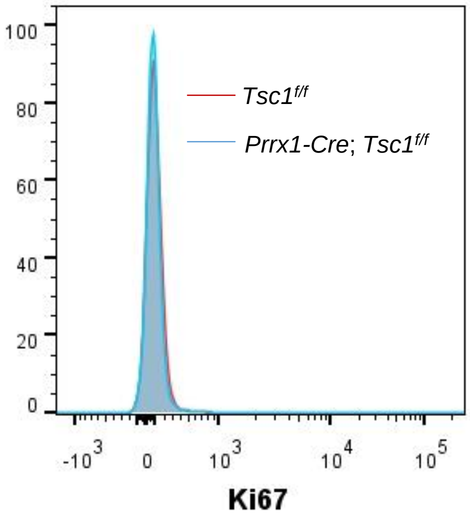

Figure S10

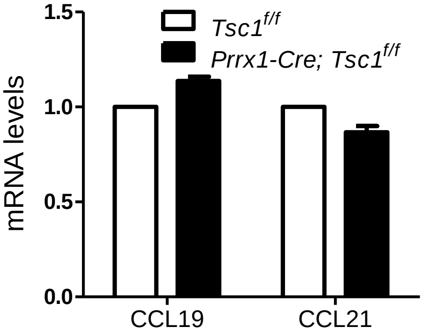

Figure S11

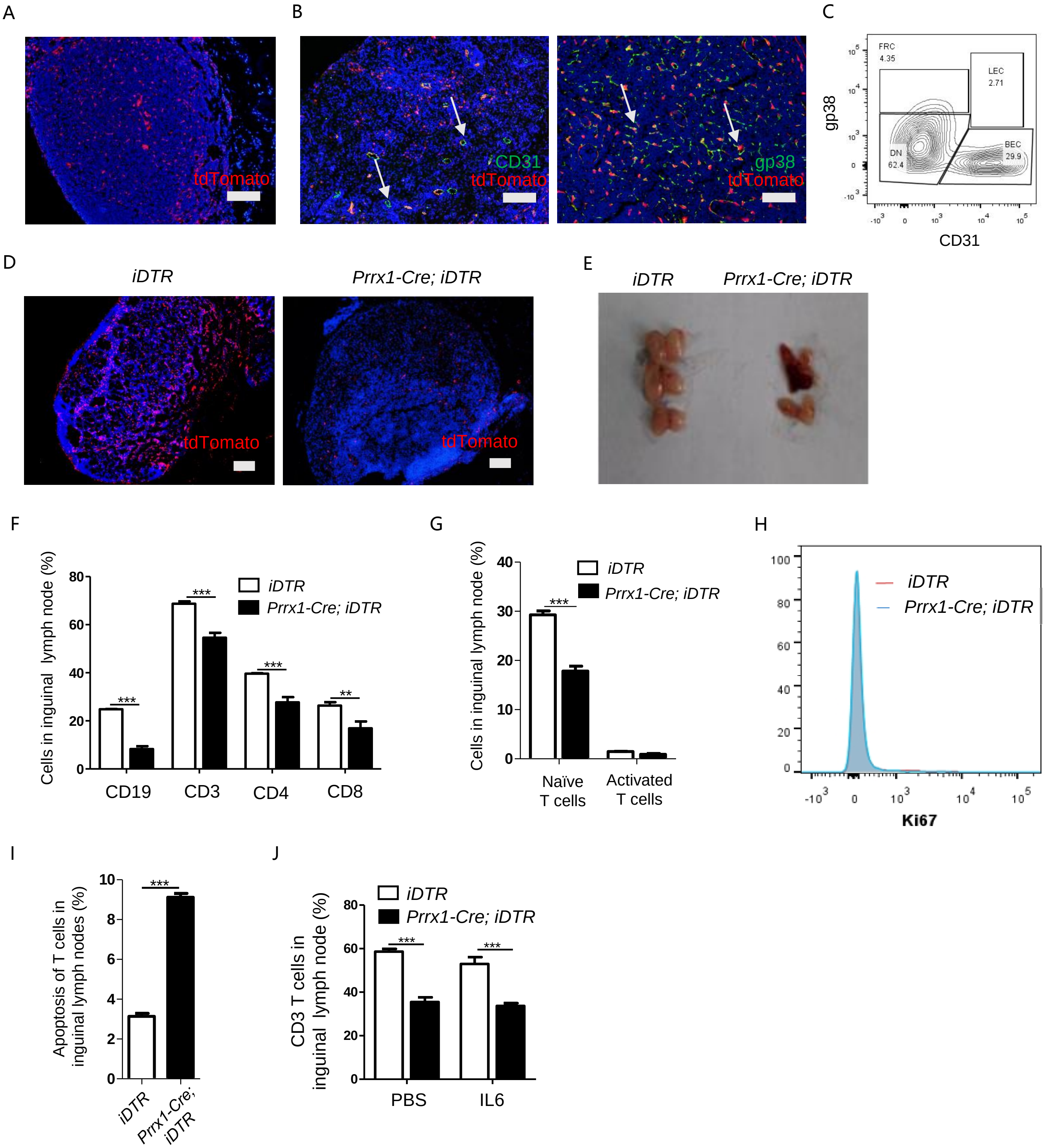
A
tdTomato
B
CD31
tdTomato
gp38
tdTomato
C
FRC 4.35
LEC 2.71
DN 62.4
BEC 29.9
gp38
CD31
D
iDTR
Prrx1-Cre; iDTR
tdTomato
tdTomato
E
iDTR
Prrx1-Cre; iDTR
F
Cells in inguinal lymph node (%)
iDTR
Prrx1-Cre; iDTR
CD19
CD3
CD4
CD8
G
Cells in inguinal lymph node (%)
iDTR
Prrx1-Cre; iDTR
Naïve T cells
Activated T cells
H
iDTR
Prrx1-Cre; iDTR
Ki67
I
Apoptosis of T cells in inguinal lymph nodes (%)
iDTR
Prrx1-Cre; iDTR
J
CD3 T cells in inguinal lymph node (%)
iDTR
Prrx1-Cre; iDTR
PBS
IL6
